# Added Value of MAPSE to Assess LV Systolic Function in Conventional Cardiac Pacing

**DOI:** 10.3390/jcm14196880

**Published:** 2025-09-28

**Authors:** Liviu Cirin, Constantin Tudor Luca, Cristina Văcărescu, Adelina Andreea Faur-Grigori, Vlad Sabin Ivan, Ciprian Dima, Roxana Buzas, Daniel-Florin Lighezan, Simina Crișan, Dragos Cozma

**Affiliations:** 1 Doctoral School, “Victor Babeș” University of Medicine and Pharmacy, 300041 Timișoara, Romania; liviu.cirin@umft.ro (L.C.);; 2 Research Center of the Institute of Cardiovascular Diseases Timisoara, 300310 Timisoara, Romania; constantin.luca@umft.ro (C.T.L.); simina.crisan@umft.ro (S.C.); dragos.cozma@umft.ro (D.C.); 3 Faculty of Medicine, Department of Internal Medicine I, Discipline of Medical Semiology I, “Victor Babes” University of Medicine and Pharmacy, 300041 Timișoara, Romania; buzas.dana@umft.ro (R.B.);; 4 Institute of Cardiovascular Diseases Timisoara, 300310 Timișoara, Romania; 5 Department of Cardiology, “Victor Babeș” University of Medicine and Pharmacy, 300041 Timișoara, Romania; 6 Center for Advanced Research in Cardiovascular Pathology and Hemostaseology, “Victor Babes” University of Medicine and Pharmacy, 300041 Timișoara, Romania

**Keywords:** MAPSE, LVEF, cardiac pacing, heart failure

## Abstract

**Background:** Mitral annular plane systolic excursion (MAPSE) is a simple and widely used M-mode echocardiographic marker of left-ventricular longitudinal function that correlates well with left ventricular ejection fraction (LVEF). Conventional chronic right ventricle (RV) pacing is associated with left ventricle (LV) dysfunction, inducing heart failure (HF) and leading to the development of pacing-induced cardiomyopathy (PiCM). The aim of this study is to ascertain the clinical usefulness of MAPSE in the assessment of LV function in patients with permanent RV pacing. **Methods:** We performed a cross-sectional association analysis, enrolling consecutive patients with pacemakers and chronic RV pacing burdens over 20% (Vp > 20%) from 2021 to 2024. All patients were assessed by standard transthoracic echocardiography (TTE) with LVEF and MAPSE among other parameters being assessed. We performed a correlation test using linear regression and plotted an ROC curve. **Results:** 409 patients (mean age = 68.7 year) were included, 225 men (55%) and 245 (59.9%) with dual-chamber pacemakers. The mean follow-up period was 18 ± 2 months, with HF incidence in the study group being 23.2%. The results showed that average, septal, and lateral MAPSE all correlate well with LVEF, but septal values seemed to provide the strongest correlation (r = 0.90, *p* < 0.001), and that a septal MAPSE cut off value of <10 mm (sensitivity 99.4, specificity 42.1, AUC = 0.89) was associated with impaired LVEF (<50%). **Conclusions:** MAPSE seems to corelate well with LVEF across the spectrum of HF in pts with chronic RV conventional pacing. Septal MAPSE shows the strongest correlation with LVEF, and a value of <10 mm is a cut-off for altered LVEF, making it a potentially useful marker of cardiac function in these pts.

## 1. Introduction

Conventional right ventricular (RV) endocardial pacing has long been the mainstay treatment of atrioventricular conduction disturbances such as atrioventricular block (AVB). Even though newer methods and techniques, such as conduction system pacing (CSP) have become increasingly popular in recent years, conventional RV pacing is still the most widely used pacing practice worldwide. Chronic conventional RV pacing, especially in patients (pts) paced from the RV apex, has historically been associated with negative effects on left ventricular function, leading to a form of heart failure (HF) called pacing-induced cardiomyopathy (PiCM). It is usually defined by most authors as left ventricular (LV) systolic dysfunction resulting from the electromechanical dyssynchrony induced by RV pacing. Definition of this condition varies among authors, and its management is still a subject of discussion [1,2]. The 2021 ESC guidelines on cardiac pacing state that PiCM seems to be more prevalent after 2–4 years of RV pacing in patients with a ventricular pacing burden of over 20% (ventricular paced percentage—Vp%) and thus sets this limit for considering interventions for pacing-induced HF. The current guidelines also affirm that this limit is supported by observational data, but that there is no actual, hard data to support that “any percentage of RV pacing can be considered as defining a true limit below which RV pacing is safe and beyond which RV pacing is harmful” [3,4]. According to one meta-analysis, more than 1 in 10 pts with chronic RV apical pacing developed PiCM. Among cited risk factors are male sex, wider native and paced QRS durations, higher RV pacing burdens, and lower baseline LVEF [5]. The optimal management strategy has yet to be defined and the ability to predict which patients are more at risk to develop PiCM is underwhelming [6]. Thus, it seems necessary to identify complementary tools that can be used to rapidly, reproducibly, and accurately assess LV function in these patients, a problem we identified in our review published in 2024 [7]. We proposed using mitral annular plane systolic excursion (MAPSE), an old M-mode-derived ultrasound parameter for assessing left ventricle (LV) systolic longitudinal function that is easy to use and correlates well with left ventricular ejection fraction (LVEF). While it is an established and validated parameter in many clinical scenarios, no evidence of its possible use in cardiac-paced patients seems to be published or validated, making such a study on a potential novel application useful.

## 2. Materials and Methods

### 2.1. Study Design

This study was designed to test whether MAPSE can be used as a reliable marker of left ventricular systolic function in patients with RV-paced rhythms. Pts with standard indications for pacing according to the 2021 ESC guidelines on cardiac pacing and resynchronization therapy (bradycardia due to sinus-node dysfunction and/or atrioventricular block) and significant ventricular pacing burdens defined as lifetime Vp > 20% were consecutively included from 2021 to 2024 [4]. The devices implanted were either single-chamber (VVI) or dual-chamber (DDD) pacemakers from a variety of manufacturers. The pacing leads used were either passive fixation or more rarely active ones, with atrial leads being usually positioned at the right atrial appendage (RAA), while RV leads were positioned either at the RV apex (RVA) or the interventricular septum (IVS). Patients with Vp ≤ 20%, other evident causes of HF (acute coronary syndrome, myocarditis, etc.), or mechanical mitral valves were excluded. Patients who fulfilled guideline criteria for cardiac resynchronization therapy (CRT) upgrade, but in whom implantation of a biventricular pacing system or conduction system pacing lead (CSP) was unsuccessful or met by patient refusal were also included due to the need to also evaluate the correlation for lower LVEF than 35%.

The study was conducted in accordance with the 1964 Helsinki Declaration, and the protocol was approved by the Ethical Review Committee of “Victor Babes” University Of Medicine and Pharmacy Timisoara (Approval Code 42/Approval Date 20 October 2023). Written informed consent was obtained from all patients included in the study.

### 2.2. Screening, Enrollment and Follow-Up

**Figure 1:**
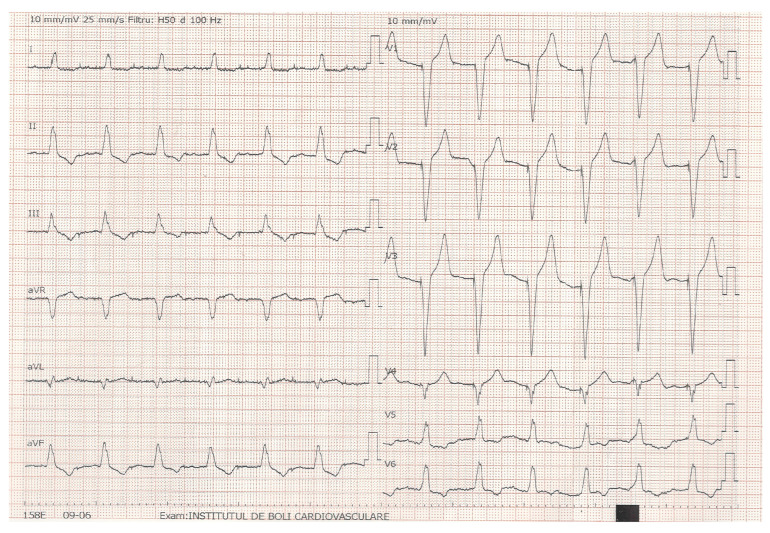
Standard 12-lead ECG during RV septal pacing.

Patients were enrolled consecutively and assigned to VVI(R) or DDD(R) pacing modes according to atrial status and guideline recommendation. Lower rate limits (LRL), night rates, rate response function, AV delays, and other proprietary algorithms such as Managed Ventricular Pacing (MVP™)—Medtronic or Ventricular Intrinsic Preference (VIP™)—Abbott were activated or deactivated, according to each patient’s needs, the device programming being tailored. Some pacing settings were altered during the study period to benefit the long-term outcome. Baseline assessments included screening for natriuretic peptides (NT-proBNP values ≥ 125 pg/mL) for HF diagnosis, standard 12-lead ECG (Figure 1), device interrogation, and transthoracic echocardiography (TTE). All patients had follow-up visits, with TTE assessment and device interrogation.

### 2.3. Echocardiographic Assessment

**Figure 2:**
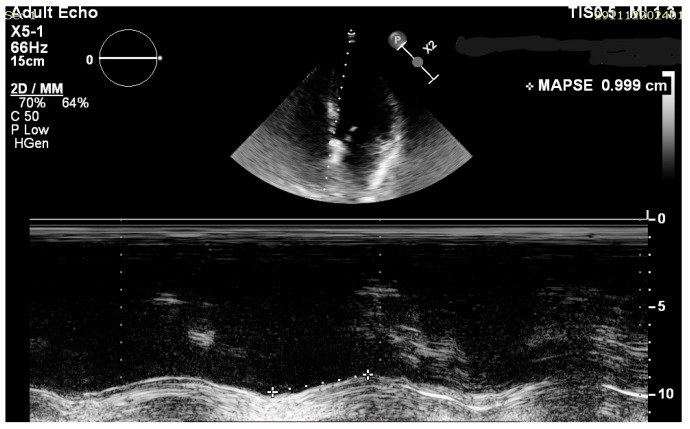
Standard A4C view used for M-mode septal MAPSE assessment.

**Figure 3:**
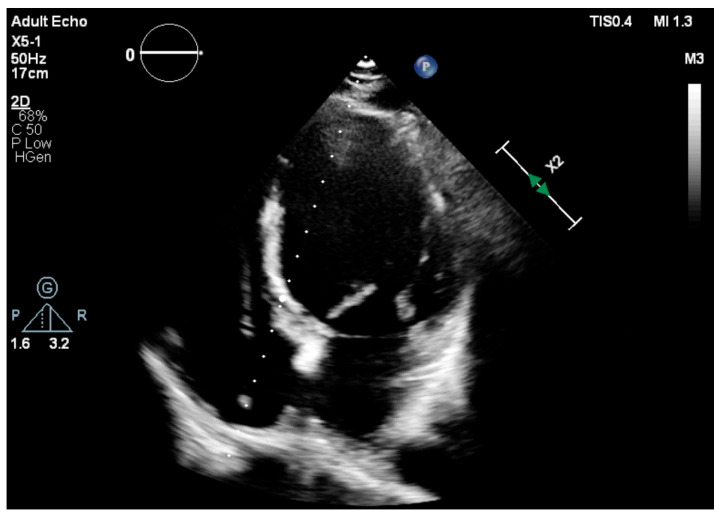
A4C view used for LVEF and MAPSE assessment; pacemaker lead present in the right chambers.

Standard transthoracic echocardiography (TTE) was performed with the use of the Vivid 7 system from General Electric (GE Healthcare Technologies, Inc., Chicago, IL, USA) or a Philips EPIQ CVx (Koninklijke Philips N.V., Amsterdam, The Netherlands) cardiac ultrasound machine. Images were obtained in the parasternal and apical views with the patients usually positioned in the left lateral decubitus position. Two-dimensional, M-mode, complete color, and spectral Doppler studies were performed in accordance with EACVI guidelines. To assess LVEF, the biplane Simpson’s method was used, and for MAPSE we used both the average value in millimeters (mm) between septal and lateral mitral annuli and the individual ones, measured in the apical four chamber (A4C) view (Figure 2 and Figure 3). Irregular rhythms (Afib, PVCs) were managed by using average values over at least 5 cardiac cycles. Images were obtained using standard manufacturer settings.

The measurements using M-mode were made parallel to the LV walls with a deviation below 15 degrees and used the distance between the peak (end-systole) and nadir (end-diastole) of the M-mode tracing [8].

All other echocardiographic parameters including LV volumes, left atrial volume index (LAVi), and Doppler and tissue Doppler variables used to determine LV diastolic function were measured, and RV function was assessed using TAPSE and RV free wall S’. The images were stored digitally and transferred to the workstation for offline analysis using dedicated proprietary software.

### 2.4. Statistical Analysis

To assess the potential correlation between the left ventricular ejection fraction (LVEF) and mean, septal, and lateral mitral annular plane systolic excursion (MAPSE) values, we used a simple linear regression test and the Pearson correlation coefficient. This test measures the linear relationship between the variables, providing a correlation coefficient (r) that ranges from −1 to 1. An r value closer to 1 or −1 indicates a strong correlation, while a value closer to 0 suggests a weak correlation. Receiver operating characteristic (ROC) curves were plotted to examine the performance of MAPSE to differentiate patients with impaired systolic function (LVEF < 50%) from those with normal systolic function, and sensitivity and specificity were determined accordingly. Relationships between quantitative values were analyzed using Deming regression, and agreement was assessed with the Bland–Altman test. Statistical analyses were performed using the Microsoft Excel Analysis ToolPak version 365 (Microsoft Corporation, Redmond, WA, USA) and SAS software version 9.4 (SAS Institute, Cary, NC, USA) with *p* values < 0.05 being considered statistically significant.

## 3. Results

In total, 409 pts with a mean age of 68.77 years old were included in our study, 55% being male. HF diagnosis was established according to ESC guideline criteria and pts were further categorized into the approved LVEF phenotypes (HfpEF-LVEF ≥ 50%, HfmrEF-LVEF = 41–49% and HfrEF-LVEF ≤ 40%). A total of 23.2% of pts were diagnosed with HF. The baseline data of the study population is presented in Table 1. Most pts with HFrEF and HFmrEF had guideline-directed medical therapy with ARNi, SGLT2 inhibitors, and MRA according to tolerances, while pts with HFpEF mostly benefited from SGLT2 inhibitors and ACEi or ARBs in absence of any contraindications. Betablockers and loop diuretics were titrated as needed and on a case-by-case manner. Table 2 shows the TTE values, while Table 3 presents the electric parameters of the study group.

59.9% of pts were carrying a dual-chamber device and 72.1% had the ventricular lead positioned at the RV apex. Slightly wider QRS complexes were noticed in the RVA-paced group.

**Figure 4:**
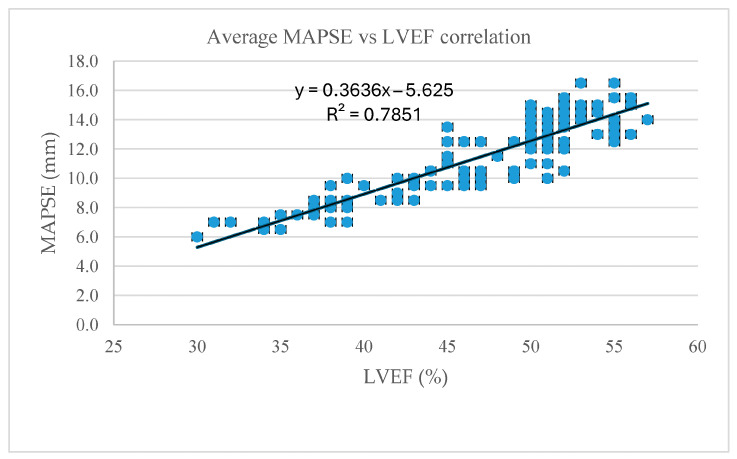
Correlation between the average MAPSE value in millimeters and LVEF in percentage.

The Pearson correlation coefficient for average MAPSE and LVEF was r = 0.89 (*p* < 0.001), thus indicating a significant positive correlation between the two parameters, making the average MAPSE value a generally good surrogate marker of LV performance in patients with paced rhythms (Figure 4). Lateral vs. septal MAPSE calculation showed a slightly better correlation with the septal MAPSE value (r = 0.88 vs. r = 0.90, *p* < 0.001), suggesting that septal MAPSE is a better surrogate marker of LV function in this subset of patients (Table 4).

**Figure 5:**
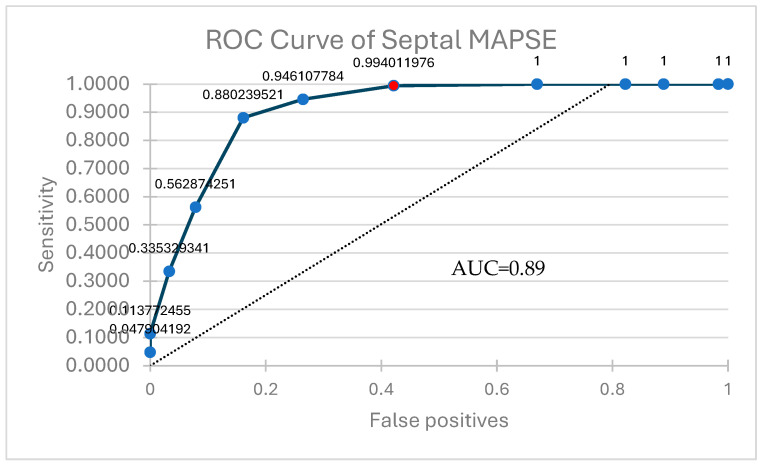
ROC curve analysis of Septal MAPSE.

We considered that, given the slightly stronger correlation with LVEF being between the septal value of MAPSE, its use as a surrogate marker of LV function would be more advisable than the lateral or average values. To further characterize it, we decided to do an ROC curve analysis to find cut-off values (Figure 5). The test revealed that a septal MAPSE < 10 mm can reliably predict an LVEF < 50% and thus HF (HFmrEF and HFrEF phenotypes specifically) (AUC = 0.89, sensitivity 99.4, specificity 42.1). Mean difference 10.78 (95% confidence interval (CI) 10.53–11.02) (Table 5).

**Figure 6:**
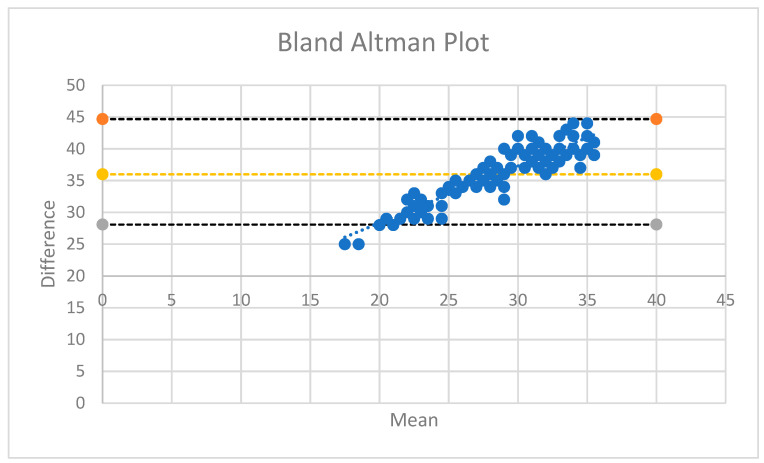
Bland–Altman plot.

**Figure 7:**
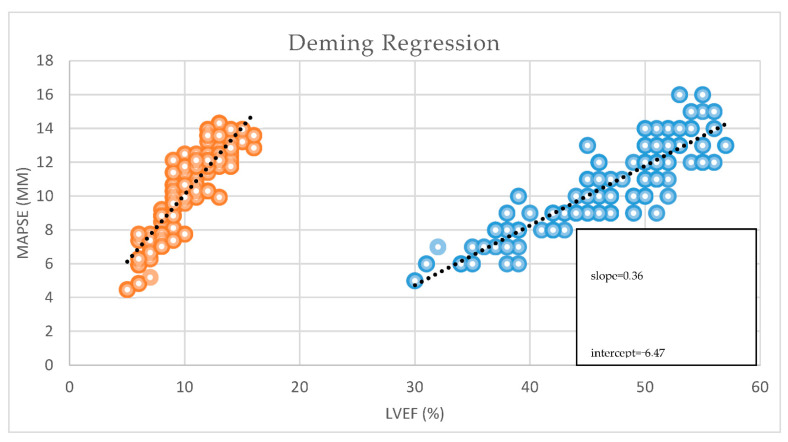
Deming regression of MAPSE and LVEF.

Figure 6 shows the Bland–Altman plot used to assess the agreement between the two measurement methods. As most values fall within the lines of agreement, the differences are clinically acceptable, as the two methods agree sufficiently (Figure 7).

We also performed a Deming regression to analyze the relationship between these two variables, accounting for measurement errors in both. It provides an estimate of the linear association, including the slope and intercept, indicating how changes in MAPSE relate to changes in LVEF. MAPSE can reliably predict or be used as a surrogate for LVEF, with the regression line illustrating the nature of their relationship.

## 4. Discussion

MAPSE seems to be a reliable and reproducible marker of LV function in the context of conventional, endocardial RV pacing rhythms, one that correlates well with LVEF, confirming a supposition that brings new clinical use for this M-mode parameter. Septal MAPSE seems to be a slightly better TTE variable than the lateral or even the average value for predicting LV systolic function in pts with paced rhythms, providing the strongest correlation with LVEF. Hence, septal MAPSE might be a simple bedside tool for helping clinicians with the early detection of LV systolic dysfunction in pts with chronic RV pacing, especially when other more complex parameters or measurements are not available or feasible. A septal MAPSE value of 10 mm or lower holds a strong correlation to altered LVEF, defined as ≤49% (HFmrEF and HFrEF). For pts with HFpEF, however, other parameters used in conjunction, such as mitral inflow pattern and tissue Doppler, might be needed for diagnosis, the correlation in this case being weaker. It seems that septal MAPSE can be used as a useful tool in screening for HF in pts with significant ventricular pacing burdens.

Longitudinal shortening of the LV contributes to approximately 60% of stroke volume, and because of the subendocardial location of longitudinal myocardial fibers, they are more sensitive to pathological processes, making any parameter evaluating their function a valuable and early tool for systolic dysfunction identification [9]. Generally, the established MAPSE cutoff values used for a normal LVEF are reported to be ≥11 mm in women and ≥13 mm for men, while for severely reduced EF, a MAPSE < 6 mm for men and women were identified. Lateral normal MAPSE values are larger (≥15 mm), while septal values tend to be slightly lower [10].

The results of this study seem to show a cut-off value for septal MAPSE of <10 mm, a lower value than that suggesting an impaired LV systolic function, with a sensitivity of 99% and specificity of 42%. These results seem to correlate positively with other previous studies, which indicate that a septal MAPSE ≥ 12 mm in pts with septic shock is linked with normal LVEF while lower values are associated with an EF < 50%, and that lateral MAPSE usually generates higher values than its septal counterpart [8].

### 4.1. Future Direction

Given that pts with PiCM or HFrEF with significant ventricular pacing burdens benefit from CRT, there is room for research in this domain, as apparently as of now, no study has been published on the use of MAPSE as a marker for CRT response in pts upgraded to biventricular pacing or de novo implanted with a biventricular device. LV-only fusion pacing is another way of achieving resynchronization in some pts by obtaining a narrower QRS by fusing intrinsic RV conduction with LV pacing. Ultrasound parameters for assessing LV dyssynchrony and response to CRT are time consuming, cannot accurately predict response rate and, except for LVEF, are currently not included in ESC guidelines as selection criteria [11]. Using a quick and simple tool like MAPSE to assess response in such pts and/or maybe even as a criterion for selection, might prove to be extremely useful in the future [12,13]. The same seems to be true for CSP, an emerging physiological alternative to RV pacing with solid guideline indication and growing evidence of providing an alternative to conventional CRT [14,15].

With the growing number of cardiac magnetic resonance (CMR) studies being performed worldwide, CMR-derived MAPSE has established itself as a powerful predictor of systolic function in a variety of pathologies [16,17]. Given the fact that all modern pacing systems are MR-conditional, we can safely assume that such a study can be performed in the future. We can also further speculate that TAPSE, a similar M-mode marker for evaluating RV function, could also be investigated and used in the clinical scenario of paced rhythms, another future research direction.

### 4.2. Limitations

The main limitation of our study is the low number of pts included in each HF category, especially the HFrEF group, as these pts usually benefit from an upgrade to a CRT device. The high sensitivity but low specificity of our threshold value results indicates that although we seem to accurately detect HF pts with altered ejection fraction, we do also tend to produce a rather high number of false positives. This limitation can be mitigated by the use of other markers such as tissue Doppler variables if available. The predominance of apical pacing in our study cohort can also affect results, as RVA pacing generates a slightly wider QRS complex than septal pacing. Further research is needed to understand if any significant difference in MAPSE values can be attributed to pacing site, as no such data currently exists. It is also worth mentioning the known limitations of MAPSE usage from the literature in certain well documented situations, such as pericardial effusions, mitral valve disease (ring calcification), left-sided prosthetic valves, and previous septal myocardial infarction (due to the localized area of abnormal motion), making its use in these clinical scenarios generally not recommended. MAPSE should be carefully applied in case of a mobile cardiac apex such as is seen in apical rocking, a motion sometimes noticed in dyssynchronous left ventricles. There is also some data suggesting that in patients with paradoxical septal motion (PSM), septal MAPSE might not only reflect LV function, but also RV abnormalities [18]. While conventional endocardial RV pacing does, in most cases, induce a left bundle branch block morphology on ECG and alter the physiological contraction of the LV by slower conduction through the myocardium than using the conduction system and thus can be a source of PSM, it seems logical to speculate that at least septal MAPSE in paced patients can be used as a marker of RV function as well, more data and studies being needed for validation in this case [19]. We also consider there is potential for its use as a marker of LV diastolic function in pts with pacemakers, since it has been proven to be applicable in other categories such as obese adults, again, further research in this area being needed [20]. We concur that it is also an age-dependent parameter, making its ultrasound-derived values for pediatric population different and necessitating adjustment for body size with normal and pathological values differing [21].

## 5. Conclusions

MAPSE represents a consistent and reproducible parameter for assessing left ventricular systolic function during conventional endocardial right ventricular pacing, showing good concordance with measurements of the left ventricular ejection fraction, especially of its septal value. Moreover, it is a straightforward and reproducible parameter that can be reliably assessed even in cases of suboptimal echogenicity or limited operator experience. This study seems to be the only attempt in recent publications to validate its use in this specific clinical scenario. Additional research, such as survival analysis and long term LVEF and HF monitoring, however, is needed to certify the potential role of MAPSE in identifying overall prognosis and if it could also be used as a predictive marker for HF in these patients.

## Figures and Tables

**Table 1:** Demographic data and prevalence of risk factors among the studied group (BMI, body mass index; HF, heart failure; HFrEF, heart failure with reduced ejection fraction; HFmrEF, heart failure with mildly reduced ejection fraction; HFpEF, heart failure with preserved ejection fraction; T2DM, diabetes mellitus; HTN, hypertension; CHD, coronary heart disease).

Baseline Characteristics	*n* (%)/SD
Male	225 (55.01%)
Female	184 (44.99%)
Age	68.77 ± 12.69
BMI	27.5 ± 3.71
HF diagnosed	95 (23.2%)
HFrEF HFmrEF HFpEF	42 (44.2%) 28 (29.5%) 25 (26.3%)
T2DM	112 (27,38%)
CHD	70 (17.11%)
HTN	233 (56.9%)
NTproBNP (mean)	1723 ± 1023 (pg/mL)

**Table 2:** Echocardiographic parameters of studied group (LVEF, left ventricular ejection fraction; MAPSE, mitral annular plane systolic excursion; LA, left atrium).

Parameter		
	Mean	SD
LVEF (%) (Simpson’s)	50.23	5.07
Avg MAPSE (mm)	13.65	2.05
Septal MAPSE (mm)	13.15	2.02
Lateral MAPSE (mm)	14.16	1.97
LA volume (mL)	78.51	30.55
LA surface (cm^2^)	30.55	6.57

**Table 3:** Electric parameters of studied group (PM, pacemaker; RVA, right ventricular apex; IVS, interventricular septum; QRSd, QRS duration).

Parameters	*n* (%)
PM type	245 (59.9%) dual-chamber
Lead position	295 (72.1%) RVA pacing
	Mean values
Paced QRS axis (°)	−45.38 RVA pacing 52.26 IVS pacing
Paced QRSd (msec)	152.37 IVS pacing
	159.12 RVA pacing

**Table 4:** Correlation test results of MAPSE and LVEF.

Parameter	Correlation (r Value)	*p*
Average MAPSE	0.89	**<0.001**
Septal MAPSE	0.90	
Lateral MAPSE	0.88	

**Table 5:** Confusion matrix.

	Predicted +	Predicted −
Actual +	249 (TP)	1 (FN)
Actual −	92 (FP)	67 (TN)

## Data Availability

The original contributions presented in this study are included in the article. Further inquiries can be directed to the corresponding author.

## Abbreviations

The following abbreviations are used in this manuscript:

MAPSE	Mitral annular plane systolic excursion
RV	Right ventricle
LV	Left ventricle
HF	Heart failure
PiCM	Pacing-induced cardiomyopathy
AVB	Atrioventricular block
CSP	Conduction system pacing
LVEF	Left ventricular ejection fraction
CRT	Cardiac resynchronization therapy
RAA	Right atrial appendage
RVA	Right ventricular apex
IVS	Interventricular septum
TTE	Transthoracic echocardiography
A4C	Apical 4 chamber
LAVi	Left atrium volume index
HFpEF	Heart failure with preserved ejection fraction
HFmrEF	Heart failure with mildly reduced ejection fraction
HFrEF	Heart failure with reduced ejection fraction
PSM	Paradoxical septal motion
